# Experimental and computational electrochemistry of quinazolinespirohexadienone molecular switches – differential electrochromic vs photochromic behavior

**DOI:** 10.3762/bjoc.15.240

**Published:** 2019-10-18

**Authors:** Eric W Webb, Jonathan P Moerdyk, Kyndra B Sluiter, Benjamin J Pollock, Amy L Speelman, Eugene J Lynch, William F Polik, Jason G Gillmore

**Affiliations:** 1 Department of Chemistry, Hope College, 35 East 12th Street, Holland, MI 49422-9000, USA

**Keywords:** cyclic voltammetry, density functional theory, heterocycles, molecular switches, photochromic photooxidants, spirocycles

## Abstract

Our undergraduate research group has long focused on the preparation and investigation of electron-deficient analogs of the perimidinespirohexadienone (PSHD) family of photochromic molecular switches for potential application as "photochromic photooxidants" for gating sensitivity to photoinduced charge transfer. We previously reported the photochemistry of two closely related and more reducible quinazolinespirohexadienones (QSHDs), wherein the naphthalene of the PSHD is replaced with a quinoline. In the present work, we report our investigation of the electrochemistry of these asymmetric QSHDs. In addition to the short wavelength and photochromic long-wavelength isomers, we have found that a second, distinct long-wavelength isomer is produced electrochemically. This different long-wavelength isomer arises from a difference in the regiochemistry of spirocyclic ring-opening. The structures of both long-wavelength isomers were ascertained by cyclic voltammetry and ^1^H NMR analyses, in concert with computational modeling. These results are compared to those for the symmetric parent PSHD, which due to symmetry possesses only a single possible regioisomer upon either electrochemical or photochemical ring-opening. Density functional theory calculations of bond lengths, bond orders, and molecular orbitals allow the rationalization of this differential photochromic vs electrochromic behavior of the QSHDs.

## Introduction

Photochromic molecular switches, in which light initiates reversible coloration of a short-wavelength isomer (SW) to a long-wavelength isomer (LW), which fades back to SW either thermally or photochemically, have become ubiquitous in a wide range of applications [1–5]. Typically, organic photochromism results from a spirocyclic ring-opening or other isomerization which results in increased conjugation. Electrochromism is also of increasing materials relevance, e.g., for self-dimming automotive mirror and aircraft window darkening applications [6–8]. In electrochromic applications, the color change is generally due to a change in the oxidation state of a conjugated system. This change in the oxidation state is most often concomitant with conformational and orbital occupancy changes, rather than any σ-bond-forming or breaking processes. The viologens are perhaps the most ubiquitous example of small molecule organic electrochromism [6–7,9].

**Scheme 1:**
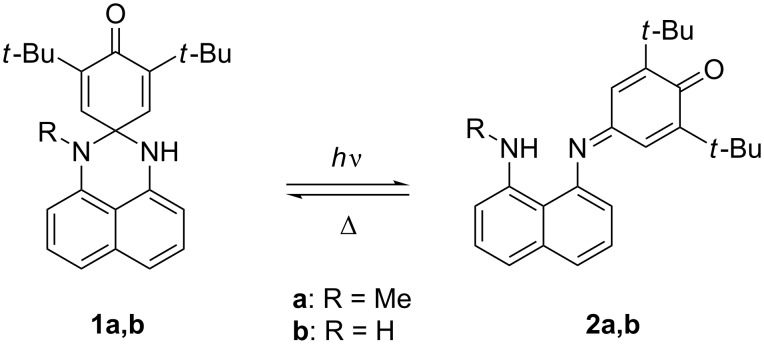
PSHD photochromism [10].

One example that combines photochromic and electrochromic behavior (the latter of an unusual sort) is the class of perimidinespirohexadienones **1** (PSHDs) whose synthesis, electrochemistry and UV–vis spectroscopy were reported by Minkin and co-workers [10] for **1a** (Scheme 1). Electrochemically, they report observing a single, two-electron reduction peak and two distinct one-electron oxidation peaks upon scanning using cyclic voltammetry. They therefore proposed [10] that the electrolysis of **1a** proceeds by an “ECE” (electrochemical-chemical-electrochemical) mechanism (**1a** → **1a^•−^** → **2a^•−^** → **2a^2−^** → **2a^•−^** → **2a**) in which the dienone portion of the molecule accepted the first electron, followed by a radical anion rearrangement to the long-wavelength isomer, whose radical anion is so much easier to reduce that it immediately accepts a second electron at this potential; on the oxidative return wave the subsequent oxidations of the LW dianion to its radical anion and then its neutral state are observed. Thus, in this unusual system, electrochromism proceeds by the same sort of spirocyclic ring-opening as the photochromic rearrangement but occurs from the radical anion rather than a photoexcited state. The reduction of the molecular switch necessary for electrochromism is in a sense catalytic: the rearranged product is reoxidized to a neutral LW isomer, which reverts thermally to SW upon standing, just as it does when the LW is generated photochemically.

**Figure 1:**
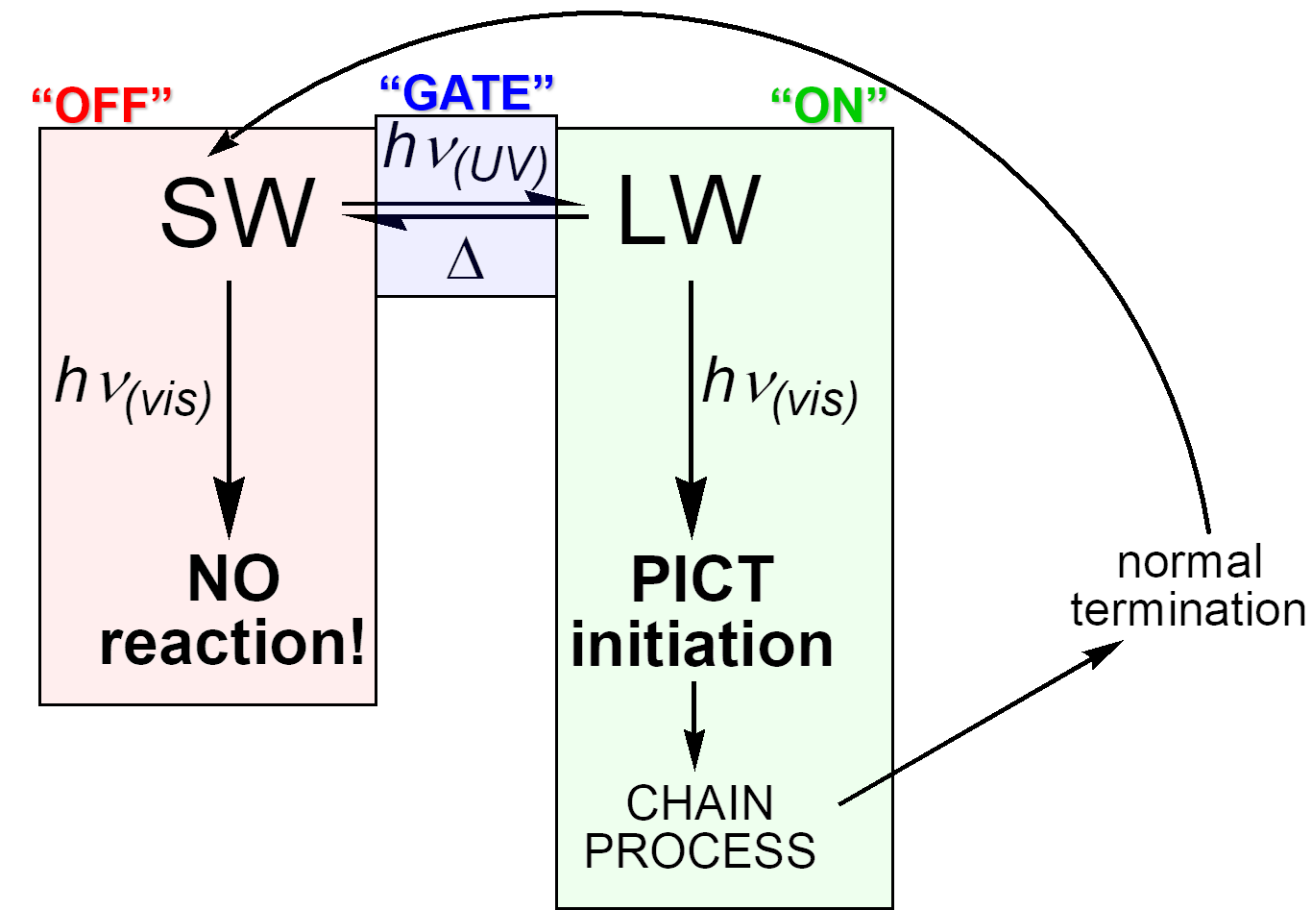
Proposed gating of sensitivity to photoinduced charge transfer by a photochromic photooxidant in which only LW is a competent photooxidant of the donor of interest.

**Scheme 2:**
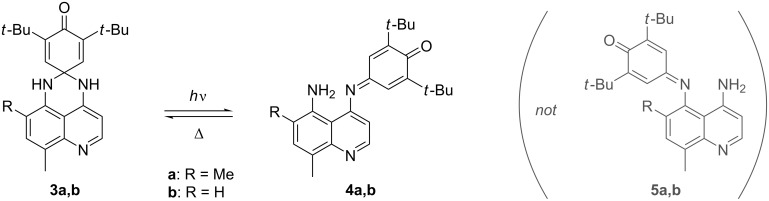
QSHD photochromism [21].

The PSHD system was of interest to us as a potential photochromic photooxidant that would add an additional level of gating to photoinduced charge transfer (PICT) initiated processes (Figure 1). PSHDs were promising for this, as their photochromic reversion of LW back to SW proceeds purely thermally, leaving the long-wavelength absorption available for bimolecular PICT. Moreover, the LW is sufficiently more reducible that, even accounting for its lower excitation energy, the LW excited state, LW*, is more reducible than SW*, making LW the more potent photooxidant of the two. However, for practical use as photooxidants, the difference in the reduction potential between LW and SW would need to be increased further, and LW would need to be more reducible to be of use in photooxidation of relevant substrates (e.g., Dewar benzenes, quadricyclanes, or bishomocubanes as quantum amplified isomerization substrates [11–15], or vinylcarbazole or alkoxystyrene derivatives for radical cation cylcloaddition and polymerization reactions [16–20]). We thus proposed the replacement of the naphthalene in **1a** with a more electron-deficient quinoline ring. Due to the saturated spirocyclic carbon insulating the dienone electrophore from the quinoline moiety in the SW form, we expected minimal change in the SW reduction potential relative to the PSHDs, but a significant difference for the completely conjugated LW isomer(s). Previously we reported the detailed synthesis of two novel quinazolinespirohexadienone (QSHD) photochromes **3a**,**b** (Scheme 2) and their photochemical properties as well as a proof of structure for the photochemically generated long-wavelength isomer (pLW) **4a**,**b** (not **5a**,**b**) [21]. Herein, we report the electrochemistry of these QSHDs.

## Results and Discussion

### Electrochemical analysis

**Figure 2:**
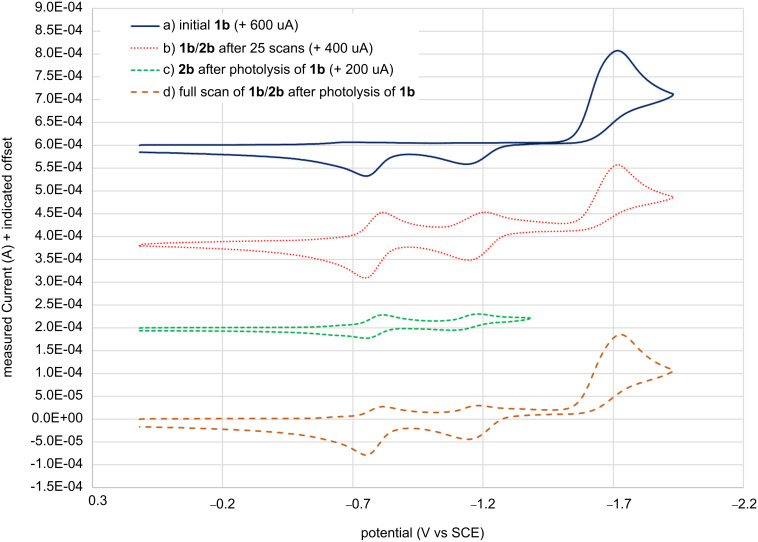
Cyclic voltammograms of a) **1b** before irradiation or electrolysis (solid blue), b) **1b/2b** after 25 scans (dotted red), c) **1b/2b** upon photolysis of **1b** (dashed green) scanned over a narrower potential window so as not to reduce **1b**, and d) **1b/2b** scanning the full potential window after photolysis of **1b** (dashed orange).

When we replicated cyclic voltammetry (CV) experiments on PSHDs, we observed similar voltammograms for both **1a** and **1b** (Figure 2), consistent with the two-electron reduction and two one-electron oxidation processes reported by Minkin for **1a** [10]. As expected, a growth of two reversible one-electron reduction–oxidation peaks was observed upon multiple scans, representing the reduction and oxidation of the electrogenerated long-wavelength form **1b** generated in situ. As expected, either photolysis or multiple CV scans led to the same LW reduction and oxidation peaks (within the error bars indicated in Table 1). We also report an identical *E*^o^_red_ of **2a** as Minkin [10], though a 140 mV difference was found in the *E*^o^_red_ of **1a**, which can be attributed at least in part to our use of half-peak potentials for all irreversible peaks, while Minkin reported peak potentials (our peak potentials were within 60 mV of Minkin’s value). We found the parent compound **1b**, whose electrochemistry was not previously reported by Minkin, to be 50 mV less reducible than **1a** (−1.68 V vs −1.63 V for **1a**), in qualitative agreement with our computational predictions [22]. Similarly, **2b** differed from **2a** by only 20 mV (Table 1) where computations predicted a minimal difference. The electrogeneration of **2b** through repeated potential step bulk electrolysis of **1b** in acetonitrile-*d*_3_ yielded a sufficient quantity of LW to obtain a ^1^H NMR spectrum, which revealed identical chemical shifts as those for the photogenerated **2b**. First and second reduction potentials were also the same, within error limits, for photogenerated and electrogenerated **2b**, as would be expected. This is consistent with the excellent overlap of all four voltammograms in Figure 2.

**Table 1:** Experimental and computational *E*^o^_red_ of **1a**,**b** and **3a**,**b** and their LW isomers, reported in V vs SCE.

		Electrochemical			Photochemical	
Compd.	Type	*E*^o^_red_ (SW)^a^	*E*^o^_red_ (eLW^·−^)	*E*^o^_red_ (eLW)	*E*^o^_red_ (pLW^·−^)	*E*^o^_red_ (pLW)

**1a**	literature^b^	−1.77	−1.325	−0.85		
	exptl.^c^	−1.628 ± 0.031	−1.220 ± 0.049	−0.857 ± 0.010		
	*predicted*^d^	−*1.56*		−*0.89*		−*0.89*
**1b**	exptl^c^	−1.681 ± 0.013	−1.202 ± 0.032	−0.871 ± 0.013	−1.170 ± 0.060	−0.865 ± 0.005
	*predicted*^d^	−*1.70*		−*0.90*		−*0.90*
**3a**	exptl^c^	−1.632 ± 0.030	−1.26 ± 0.11	−0.866 ± 0.009	−1.029 ± 0.035	−0.730 ± 0.005
	*predicted*^d^	−*1.61*		−*0.88*		−*0.72*
**3b**	exptl^c^	−1.631 ± 0.017	−1.069^ad^ ± 0.029	−0.843 ± 0.012	−1.008 ± 0.024	−0.729 ± 0.004
	*predicted*^d^	−*1.63*		−*0.86*		−*0.72*

^a^Irreversible peak. *E*^o^_red_ is reported as the half-peak potential *E*^p/2^_red_ (except in literature value for **1a**). ^b^Reference [10]. Irreversible SW peak reported as peak potential (*E*^p^_red_), not half-peak potential (*E*^p/2^_red_). ^c^Experimental values in CH_3_CN containing 0.1 M Bu_4_NPF_6_, standardized vs ferrocene/ferrocenium and corrected to vs SCE as in reference [23]; error bars = one standard deviation from the mean of at least 7 replicates. ^d^Computational *E*^o^_red_ predicted using correlation 6 in reference [22] based on the energies of the corresponding ground-state and one-electron-reduced species computed using B3LYP/6-31G(d) with implicit acetonitrile solvent using the CPCM solvent model with UAKS radii, on geometries optimized in the gas-phase; predictions of second reduction potentials are not possible by this method.

The ECE mechanism reported by Minkin for the electrochromism was further supported by bulk electrochemical experiments. With repetitive conditioning and scanning under argon-deaerated conditions, the initially yellow **1b** solution turned to an orange-red color, which we hypothesized to be the LW dianion, **2b^2−^**. Upon exposure to air this solution immediately turned green, the known color of the LW isomer **2b**. The addition of benzoquinone (a chemical oxidant) to the electrochemically reduced solution under argon gave similar results, consistent with our hypothesis. When the yellow solution remained open to the atmosphere during electrolysis, the initially yellow solution turned green at first (presumably while air oxidation of **2b^2−^** to **2b** could keep up with electrochemical reduction). With further electrolysis even these solutions turned to the orange-red color observed for the deaerated solutions. The solutions did become green upon prolonged exposure to air. This behavior seems indicative of insufficient transport of air into the cell through the small vents in the cell cap to replace the oxygen being consumed during repeated electrolytic scans. Similar results were observed for solutions of **3b** suggesting a similar ECE mechanism to that of **1a** is likely also occurring for **3b**.

**Figure 3:**
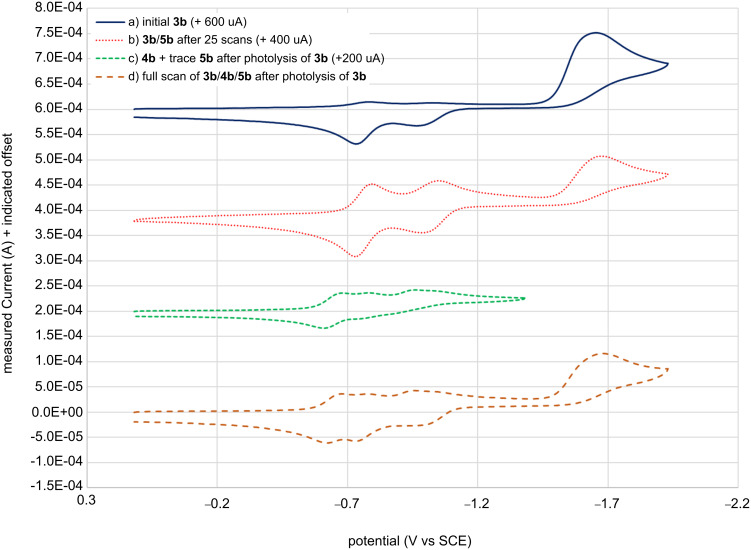
Cyclic voltammograms of a) **3b** (with trace **5b**) before irradiation or electrolysis (solid blue), b) **3b** + **5b** after 25 scans (dotted red) showing the growth of the two **5b** reduction peaks, c) **3b** (with trace **5b**) after photolyzing but without reducing **3b** in the electrolysis (dashed green) which shows the two initial eLW **5b** and two new pLW **4b** reversible redox waves observed, and d) **3b** + **4b** + **5b** observed by scanning the full potential window after the photolysis of **3b** (dashed orange).

Cyclic voltammetric analysis of quinazolinespirohexadienone (QSHD) **3b** (Figure 3) was qualitatively similar to the parent PSHDs **1a**,**b** as expected based on structural similarity and computationally calculated molecular orbital diagrams (Figure 6 and Supporting Information File 1). Surprisingly, the *E*^o^_red_ of the electrochemically generated LW form of **3a** was more negative by 10 mV than that of **2a**, even with the more electron-deficient quinoline ring. Presumably this is because the 5-position on the benzene ring of the quinoline, is the least withdrawing point of attachment, and the inductive withdrawing properties of the quinoline nitrogen are far enough removed from the electrophore to not cause any appreciable change in reduction potential. The other qualitative difference for **3b** was the presence of two LW reduction peaks (LW → LW^•−^ → LW^2−^) on the first scan (Figure 3a, dotted red). However, the observation of the two LW reduction peaks was consistent with UV–vis spectroscopy that indicated a significant amount of a LW isomer upon solvation, and repetitive electrochemical scans exhibited the anticipated growth of the LW reduction–oxidation peaks as more of the LW isomer was generated electrochemically (Figure 3b, solid blue).

Most surprisingly, when **3b** was photolyzed to form **4b** in solution under similar conditions to our previous report [21] and then analyzed electrochemically (Figure 3c and d, dashed green and orange), four reduction (and oxidation) peaks in the region of the LW isomer were observed. Two reduction–oxidation couples matched the potential of the electrogenerated LW form observed earlier while the other two peaks were shifted more positive by 60–110 mV indicating the presence of a third electroactive species in addition to **3b** and photogenerated **4b**. Given the asymmetric nature of **3b**, two distinct options for spirocyclic ring-opening exist, leading to **4b** or **5b**. Thus, we postulated that the electrogenerated LW form was in fact **5b**.

**Figure 4:**
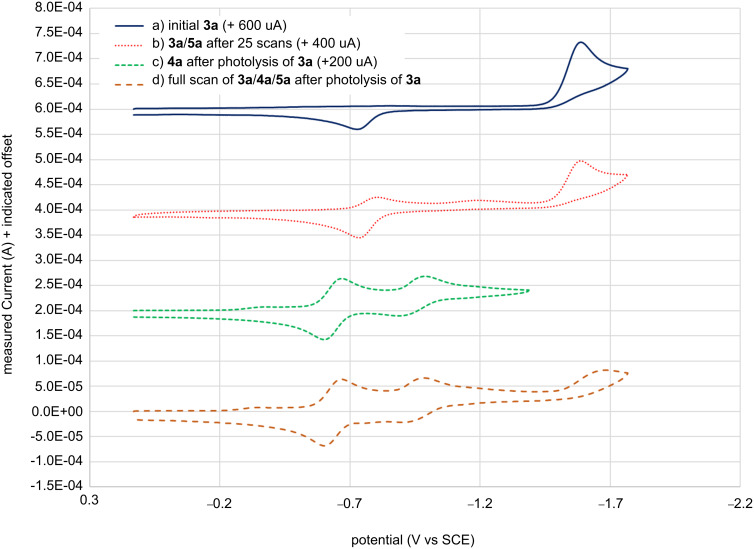
Cyclic voltammograms of a) **3a** before irradiation or electrolysis (solid blue), b) **3a** + **5a** after 25 scans (dotted red) showing the growth of the two eLW **5a** reduction peaks, c) **3a** after photolyzing but without reducing **3a** in the electrolysis (dashed green) which shows the two different reversible redox waves observed for pLW **4a**, and d) **3a** + **4a** + **5a** scanning the full potential window after the photolysis (dashed orange).

Having found two distinct LW forms depending on generation by photolysis or electrolysis of SW **3b**, we turned our attention to whether a similar phenomenon was observed for **3a**. Indeed, different redox peaks were observed in the same general LW region for the CV of photolyzed versus electrogenerated LW forms of **3a**, consistent with the electrogenerated formation of **5a** compared against the known formation of **4a** via photolysis. The voltammograms of **3a** (Figure 4) did however differ from both **3b** and **1b**. A (presumably two-electron) reduction peak was observed for **3a** but only one return oxidation wave was observed. Upon repeated scanning the two one-electron reductions of **5a** → **5a^•−^** → **5a^2−^** were observed, but still only a single (likely two-electron) return oxidation peak was observed, possibly indicating a large overpotential for the oxidation of **5a^2−^**. It is possible that **3a** undergoes only a one-electron reduction and rearrangement of **3a** → **3a**^•^**^−^**→ **5a**^•^**^−^** (without further reduction to **5a^2−^**) and subsequently only one oxidation to **5a**. But this would make the second one-electron reduction (thought to be **5a^•−^** → **5a^2−^**) observed on repeated cycling unexplainable. A more likely explanation is that the two-electron ECE reduction of **3a** still occurs to give **5a^2−^** but that the slight electron-donating nature of the additional methyl group destabilizes **5a**^•^**^−^** enough to require a substantial overpotential for reoxidation of **5a^2−^** to **5a**^•^**^−^**, such that it occurs at the same potential as oxidation of **5a**^•^**^−^** back to neutral **5a**. This could occur either sequentially at the same potential or in a single two-electron process. The latter explanation seems qualitatively in better agreement with the observed voltammogram in Figure 4.

In terms of achieving more potent photooxidants through the exchange of the naphthalene ring of the PSHDs for the more electron-deficient quinoline in the QSHDs, we had expected the LW isomers to become more easily reducible, with minimal change in reduction potential for the SW isomers. Indeed *E*^o^_red_ of the SW isomers **1b**, **3a**, and **3b** were the same within the error, and only 50 mV more reducible than **1a**. Comparing the *E*^o^_red_ of pLW isomers (**4a**,**b** relative to **2a**,**b**), a roughly 140 mV difference is observed, with **4** being more reducible than **2** as expected. However, the difference in reduction potential between PSHD and QSHD for eLW was much less. For **5b** *E*^o^_red_ was 28 mV more positive than **2b** indicating that **5b** is a slightly better oxidant than the parent PSHD. But *E*^o^_red_ of eLW **5a** was surprisingly shifted 10 mV more negative than PSHD LW **2a**, meaning it was actually harder to oxidize. Ultimately *E*^o^_red_ of **2a**, **2b**, **5a**, and **5b** are essentially the same within error limits. Apparently, the quinoline is not nearly as electron withdrawing when linked through the benzo ring as when it is linked through the heteroaromatic ring.

### Spectroscopic analysis

**Figure 5:**
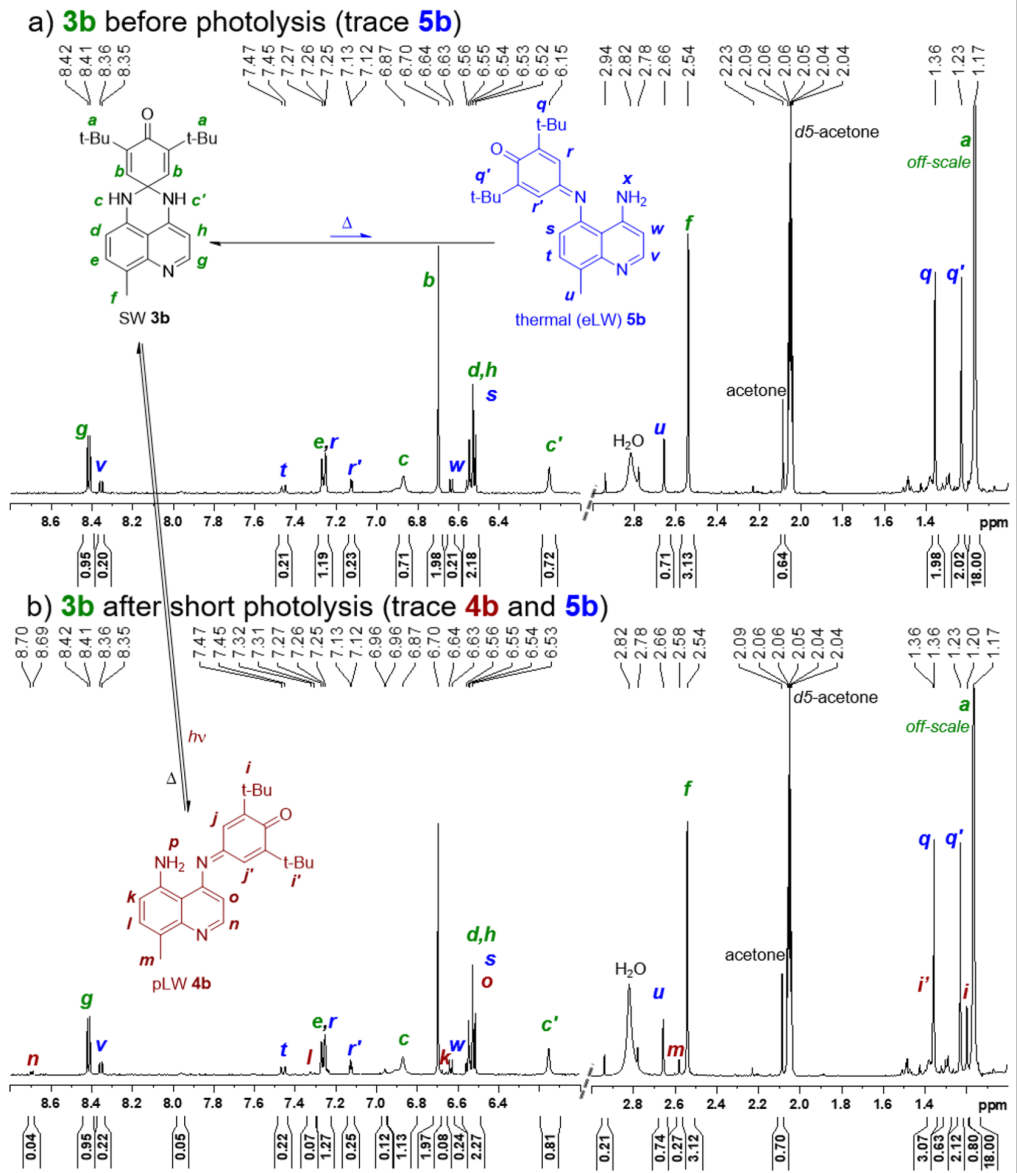
^1^H NMR distinction between SW **3a**, thermal/eLW **5a**, and pLW **4a**, in acetone-*d*_6_, as observed a) before and b) after photolysis.

The potential for two distinct products from electrolysis or photolysis as indicated through electrochemical analysis was further supported through NMR. Previous work [21] had conclusively shown through ^1^H NOE NMR spectroscopy that the LW isomers resulting from photolysis of **3a** and **3b** were **4a** and **4b**, which open toward the more electron-deficient heteroaromatic ring of the quinoline and away from the R group. Unfortunately, efforts to obtain a sufficient quantity of the electrogenerated LW form of **3a** (i.e., **4a** or **5a**) for definitive ^1^H NMR spectra or NOE experiments were not successful However, both UV–vis and electrochemical measurements indicated small amounts of a long wavelength form present in the dark immediately upon solvation (i.e., a thermal or solvatochromic LW form), particularly in **3a**. The presence of a LW isomer prior to irradiation or electrolysis was also consistent with the ^1^H NMR spectrum of a sample of **3a** which, while known to be pure in the solid state [21]), in solution showed the expected **3a** chemical shifts but also smaller peaks (ca. 20% relative to **3a**) with similar splitting and chemical shifts as those for photogenerated **4a** (to which we initially erroneously attributed them [21]). However, the frequencies for this solvatochromic LW were slightly but distinctly different from either **3a** or photogenerated **4a** (Figure 5a, e.g., consider protons **n**, **g**, and **v**). Signals for **4a** began to grow in with even very brief photolysis (Figure 5b), intentionally taken to low conversion (ca. 8% relative to **3a**) to enable comparison to the solvatachromic LW (ca. 20% relative to **3a**). Moreover, as the oxidation and reduction peaks associated with eLW **5a** (Figure 3b, dotted red) grow with increasing numbers of CV scans and match those present initially in solution in small amounts thermally (Figure 3a, solid blue), we conclude the electrogenerated and thermal LW forms are the same isomer **5a**, while it is the pLW isomer **4a** that grows in upon photolysis (Figure 3c, dashed green; Figure 5b). Furthermore, modest NOE enhancements can be observed even on the small amounts of **5a** present in these solutions. These NOEs, while weak, did aid in assigning the peaks as labelled in Figure 5b and demonstrate that the blue-labelled eLW peaks in Figure 5 are indeed on the same molecule, and that this is distinct from pLW **4a** as studied previously by NOE [21]. Thus, NMR, while not conclusive on its own, was able to provide additional support for our structural assignment.

UV–vis spectra taken of eLW solutions generated by bulk electrolysis in acetonitrile and acetonitrile-*d*_3_ were compared against photoirradiated (pLW) solutions. No difference was observed in the UV–vis spectrum of the LW isomer prepared from either photolysis or electrolysis of solutions of **1b** (as expected due to symmetry). The λ_max_ for the **2b** photogenerated solution was at 574 nm in acetonitrile and acetonitrile-*d*_3_. The electrogenerated **2b** λ_max_ at 573 nm in both solvents differed by only 1 nm, within error limits of our instrumentation. For **4a**/**5a** the λ_max_ in acetonitrile-*d*_3_ was 539 nm for the photolyzed solution (**4a**) versus 529 nm for the electrolyzed (**5a**). Similarly, for **4b** and **5b**, the photolyzed λ_max_ in acetonitrile was 558 nm versus 549 nm for the electrogenerated and was 564 nm versus the electrogenerated 550 nm in acetonitrile-*d*_3_. The differences in the UV–vis spectrum of about 10 nm between eLW and pLW isomers of QSHDs **3** indicated a similar length for the conjugated system but a significant enough difference to indicate different species. This is consistent with the formation of two constitutional LW isomers that are structurally similar yet distinct, as the difference in absorbance would be expected to be noticeable but not large. Observation of no difference in the UV–vis spectrum of the LW isomer prepared from either photolysis or electrolysis of solutions of **1b** (able to only form one LW isomer, **2b**) supports the formation of two different LW isomers for **3a**,**b** rather than attributing the small spectral changes to the presence of electrolyte in the electrolyzed solutions, to an interaction with air, or to a side reaction in solution. The shorter wavelength for electrogenerated **5a**,**b** may result from slightly less planarity and decreased conjugation versus the photogenerated **4a**,**b**. The additional *ortho*-methyl group in **5b** may exacerbate this sterically, and/or could contribute an inductively donating effect.

Finally, the thermal reversion of eLW **5a**,**b** was visually and spectroscopically obvious to have begun within a few minutes, and to be complete within 12–18 hours. This is similar to what we previously reported for pLW **4a**,**b** [21]. This comparatively slow and purely thermal reversion is consistent with the need for a thermodynamically unfavorable intramolecular (or solvent or adventitious acid/base-mediated) proton transfer to begin the reversion mechanism, as Minkin has described [10]. Interestingly, reversion of either LW species to SW is observed immediately upon removal of solvent, which played a role in complicating our analysis, as the LW species cannot be isolated as solids.

### Computational analysis

As shown above, computationally predicted reduction potentials [7] were in very good agreement with those determined experimentally (Table 1). The mean absolute deviation between computational prediction and experimental measurement of *E*^o^_red_ for all SW and LW structures in Table 1 is just 21 mV (27 mV root mean squared deviation), not far from the 14 mV mean standard deviation in the experimental measurements. This demonstrates that the computational correlation employed is useful in structural assignment and accurate within a standard of error of the experimental reduction potentials of these spirohexadienones’ different constitutional SW and LW isomers.

**Figure 6:**
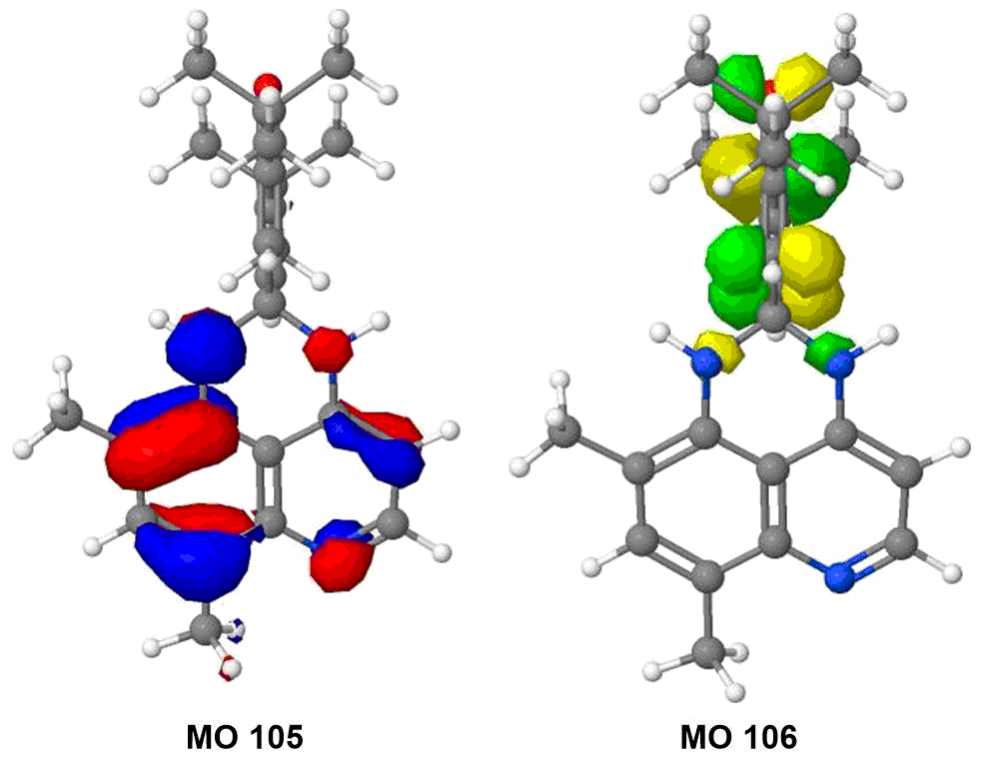
HOMO (MO 105, red and blue) and LUMO (MO 106, green and yellow) computed for **3a** in its ground (S_0_) state. Similar results were observed for the frontier orbitals of **1b** and **3b** (see Supporting Information File 1).

Density functional theory (DFT) calculated molecular orbitals (MOs, e.g., Figure 6 and Supporting Information File 1) indicate that the LUMO (MO 106 for **3a**) lies exclusively on the dienone moiety both in **1b** and in the quinazolinespirohexadienone photochromes **3a**,**b**. Thus, the dienone moiety is likely the electrophore in the SW in all PSHD and QSHD switches, just as Minkin [10] specifically asserted for **1a**. These diagrams also suggested to us that QSHDs **3** might also undergo an ECE mechanism similar to PSHDs **1**, as we have now confirmed experimentally.

**Scheme 3:**
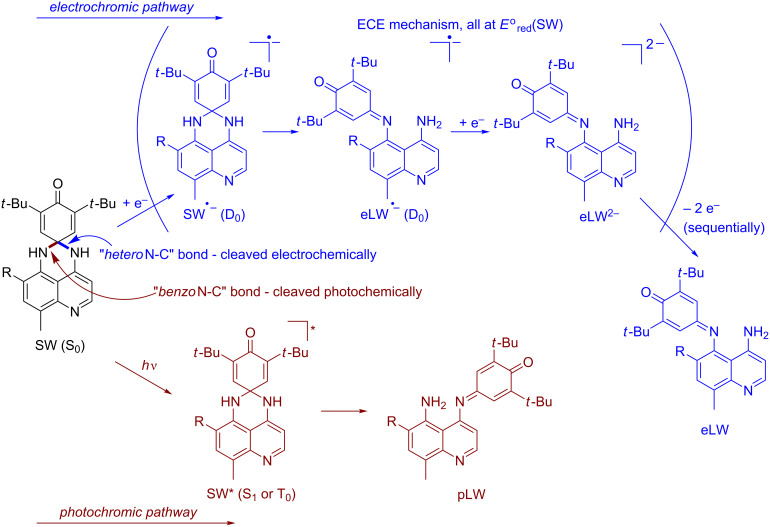
Proposed mechanism for differential formation of pLW (**4**) and eLW (**5**) from SW (**3**).

Having conclusively demonstrated the differential regiospecificity of photochromic vs electrochromic ring-opening of the QSHDs (Scheme 3) by experiment, we next sought to understand *why* SW would open differently upon excitation to SW* vs reduction to SW**^•−^**. We turned to computation (of **3a**, **4a**, and **5a**) for an explanation. Due to large differences in the methods for calculating excited and ground-states, the triplet T_0_ state was used instead of the singlet S_1_ state as the photochemical intermediate (SW*) for computational purposes. Since T_0_ is the lowest energy triplet state, it is amenable to a ground-state computation. While the real SW* photochemical intermediate may likely be the first singlet excited state (S_1_), T_0_ and S_1_ possess equivalent orbital occupancy and differ only in their spin state. Our rationale for using T_0_ rather than S_1_ is that ignoring electron-exchange interaction introduces substantially less error than using different computational methods when comparing orbitals of SW* to those of SW and SW**^•−^**. Using S_1_ for SW* would require unbalanced ground and excited-state calculations, e.g., time-independent and time-dependent density functional theories, or single-reference and multi-reference methods. However, using T_0_ for SW* is a straightforward ground-state calculation, as are the calculations of D_0_ (the radical anion) and S_0_.

**Figure 7:**
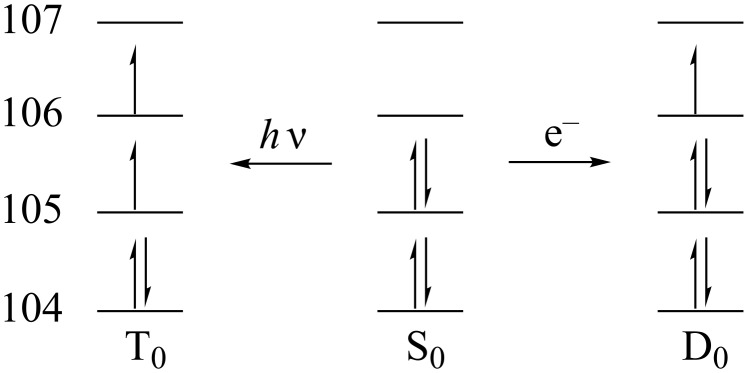
Frontier orbital occupancies of relevant electronic states of **3a**. Note: the photochemical excited state may be T_0_ (as pictured) or perhaps more likely S_1_ (inverted spin in molecular orbital 106 relative to T_0_), but T_0_ is more computationally tractable and is equivalent to S_1_ for orbital occupancy arguments.

Clearly, as shown in Figure 7, the difference in reduction vs excitation is the occupancy in what was formerly the HOMO of SW. Because S_0_ is the common precursor to both T_0_ and D_0_, analysis of the S_0_ MOs was used to explain differences in the electron distributions that lead to two different products upon either excitation to form SW* (yielding pLW **4**) or reduction to form SW**^•−^** (yielding eLW **5**). This approach of using the S_0_ MOs was validated computationally, as we found there is little change in the character and relative energies of the MOs among S_0_, T_0_, and D_0_. The calculated isosurface generated for the S_0_ highest occupied molecular orbital (HOMO), MO 105, and lowest unoccupied molecular orbital (LUMO), MO 106, of **3a** are displayed in Figure 6. MO 105 allows for a direct comparison of the T_0_ and D_0_ electron distributions, as these two states differ only in the population of this orbital. There is more contribution from this MO to the N–C bond (hereafter designated the *benzo*N–C bond, cf. Scheme 3) of the nitrogen on the benzo ring of the quinoline moiety than the N–C bond of the nitrogen off the heterocyclic ring of the quinoline moiety (hereafter referred to as the *hetero*N–C bond, cf. Scheme 3). This asymmetrical MO contribution is consistent with experimental observations. The absence of an electron from this orbital 105 in the case of T_0_ results in the preferential weakening of the *benzo*N–C bond over the *hetero*N–C bond. The formation of pLW from SW (T_0_) results from the breaking of this weaker bond. More electron density in the *benzo*N–C bond in orbital 105 might possibly suggest a stronger bond when the orbital is fully filled (as in D_0_), and could suggest that the *hetero*N–C bond might be the more likely to rupture upon reduction to SW**^•−^**.

It is common to think of thermal ring-opening as looking more like that of the biradical (T_0_ or S_1_) than of the radical anion (D_0_), so we might have presumed the structure of any thermal or solvatochromic LW isomer that occurred to have been that of pLW (**4**). However, the same rationalization that more electron density in the *benzo*N–C bond in orbital 105 might possibly suggest a stronger bond relative to the *hetero*N–C bond when the orbital is fully filled in S_0_ is how we rationalize our observation that it is eLW (**5b**) that occurs in the only case where we see a purely thermal or solvatochromic cleavage. In turn we can then rationalize on the basis of sterics (R = Me vs H) that it is logical that this thermal or solvatochromic rupture might only be observable in **3b** (R = H) but not **3a** (R = Me). This is in good agreement with our experimental observations.

Our rationalization based on molecular orbitals was confirmed by looking at both bond lengths and bond orders of the two relevant N–C bonds in SW**^•−^** and SW*. Bond order calculations provide a more quantitative explanation for the experimental observation. Because bond stability correlates to bond order, it is expected that the N–C bond which breaks upon forming the LW product would possess a smaller bond order. The BO calculations for intermediate states T_0_ and D_0_ indeed support this prediction. Table 2 summarizes the results of BO calculations for both bonds in these states. Since geometry plays a critical role in the stability of bonds, only BO calculations done from optimized geometries for each state are displayed. As shown in Table 2, *hetero*N–C has a greater bond order than *benzo*N–C in T_0_, while the relative magnitude of these bond orders is reversed in D_0_. This is consistent with the formation of eLW **5a** from **3a^•−^** D_0_ (breaking the *hetero*N–C bond) and pLW **4a** from SW* 3a*, modelled as T_0_ (breaking the *benzo*N–C bond) as shown in the proposed mechanism in Scheme 3. Finally, bond lengths were derived from geometry optimizations for D_0_ and T_0_ with and without solvent and are also included in Table 2. There is an obvious inverse relationship between calculated bond orders and bond lengths. For D_0_, a longer bond length is observed for *hetero*N–C while a longer bond length is observed for *benzo*N–C in T_0_. These results, regardless of solvent or gas-phase considerations, are consistent with the discussion above. In the cases of both D_0_ and T_0_, the longer bond is the one broken in the isomerization of SW **3a** to eLW **5a** and pLW **4a**, respectively. Similar results are also observed for S_0_. Though the differences are much more modest in S_0_, the *hetero*N–C bond is computed to be both longer and weaker, as in D_0_, and thus it makes sense that any thermal or solvatochromic LW is the eLW isomer.

**Table 2:** Computed bond lengths and bond orders of the relevant N–C bonds for photochromic (T_0_) and electrochromic (D_0_) ring-opening of **3a**. Bolded (longer and weaker) bond is the one that is cleaved in each case.

State	Bond	Bond order	Bond length (Å)	Consider for

S_0_	*benzo*N–C	0.9425	1.478	thermal LW/
S_0_	***hetero*N**–**C**	**0.9346**	1.478	solvatochromism

D_0_	*benzo*N–C	0.8879	1.508	electrochromism
D_0_	***hetero*N**–**C**	**0.8738**	**1.513**	

T_0_	***benzo*N**–**C**	**0.8143**	**1.546**	photochromism
T_0_	*hetero*N–C	0.8834	1.503	

## Conclusion

While moving from PSHD (**7**) to QSHD (**3**) increased Δ*E*^o^_red_ between SW and pLW isomers, and therefore capacity to gate photoinduced charge transfer, by about 130 mV, the excited state reduction potential E*_red_ of pLW **4** remains < +1.0 V vs SCE, insufficient to oxidize most donor molecules of relevance to materials applications. If our photochromic photooxidants are to be effective in real systems, the pLW will need to be made more electron deficient. Our computational predictions of the reduction potential [22], unfortunately not completed until after preparing the present system, were our first insight into the fact that electrochemical ring-opening was not yielding the structure we had previously proven for the photochromic pLW. We have thus demonstrated these computation’s practical utility in a real-world experimental situation, as well as their suitably high degree of accuracy. We are therefore hopeful they can help guide our search for practical photochromic photooxidants, with a more reducible pLW*.

For now, our team of undergraduate researchers has conclusively demonstrated differential regiospecificity in the photochromic vs electrochromic spirocyclic ring-opening of these QSHD molecular switches to two different LW isomers. This may provide an interesting structural framework for molecular logic or other applications that complements the recently reviewed photoelectrochromic properties observed in a range of photochromic families upon the electrochemical oxidation of either their SW or pLW isomers [24]. We have also demonstrated that any modest amount of thermal (or solvatochromic) coloration of the QSHDs is due to small amounts of the electrochromic eLW isomer **5**, rather than the photochromic pLW (**4**) as we had previously surmised [21]. Finally, we have been able to rationalize these results computationally on the basis of bond lengths, bond orders, and molecular orbital occupancy.

## Experimental

### Materials

Acetonitrile was of the highest HPLC grade (used as received or dispensed through a nitrogen-purged MBraun MBS-SPS 07-299 solvent purification system) or highest anhydrous grade (used as received). Acetonitrile-*d*_3_ and acetone-*d*_6_ were used as received in 1 g ampules (Cambridge Isotope Labs). Ferrocene, tetrabutylammonioum hexafluorophosphate (TBAH), and silver nitrate were of electrochemical grade and used as received. Photochromes **1a**,**b** were prepared according to the literature [10]. Photochromes **3a**,**b** were prepared as we previously reported [21].

### Instrumentation

Photochemical irradiations and UV–vis spectroscopy were performed as previously described [21] on 3 mL or 4 mL argon-purged acetonitrile solutions of 0.1 M TBAH supporting electrolyte and 1–5 mM analyte, sufficient to attain signals much greater than background over the full potential window considered.

Cyclic voltammetry was performed on a CHI Model 604a electrochemical analyzer or a BAS epsilon e2 electrochemical analyzer. (The equivalent BAS or CHI cells and electrodes could be used on either potentiostat interchangeably, with or without the BAS C3 cell stand.) Solutions were placed in a glass cell and bubbled with argon to deaerate for 3–5 minutes. Scans were taken under an argon blanket using a glassy carbon working electrode, a platinum wire counter electrode, and a separately sparged Ag/AgNO_3_ (10 mM) nonaqueous reference electrode isolated from the working compartment with a Vycor frit. In some cases, a bare Ag wire was used as a pseudo-reference instead. The working, reference, and counter-electrodes were arranged in a triangle through a Teflon cell cap. Rather than relying on either the Ag/AgNO_3_ reference or Ag wire pseudo-reference, the voltammetry was corrected to a ferrocene external reference. Ferrocene solutions were cyclically scanned before and after each analyte experiment, beginning in the positive (oxidative) direction over an 800 mV window roughly centered on the reduction of Fc/Fc^+^ redox couple at a scan rate of 0.1–0.5 V/s, 10^−4^ A sensitivity, and 1 mV sample interval. Internal resistance (iR) compensation was manually set to 95–99.5% of the measured resistance so that the peak separation for the reversible oxidation–reduction of ferrocene had a peak separation of ca. 60 mV and good peak shape without entering oscillation. Typical iR compensation in acetonitrile ranged from 150–230 Ω. Photochromic solutions were cyclically scanned beginning in the negative (reductive) direction at 0.1–0.5 V/s with 1 mV sample interval over an appropriate range of potentials and current sensitivity to observe the redox couples of either SW and LW or just LW isomers as desired, without reaching the solvent breakdown limit. Reduction potentials were taken as the half-peak potential of irreversible peaks or the midpoint of reversible peaks, standardized versus ferrocene (measured before and after each analyte sample), and corrected to versus SCE by adding 0.38 V [23]. At least seven replicates of each data point were obtained, with the mean value reported with error bars indicating the standard deviation from the mean among all replicates.

Potential step bulk electrolyses were performed on 3 mL argon-purged acetonitrile or acetonitrile-*d*_3_ solutions of 2 mM analyte and 0.1 M TBAH. The solution was placed in a glass cell prepared by cutting the top off a 7 mL scintillation vial and fireglazing the edges. The outer rubber of a 19/22 septum was removed with a razor blade and used to cap the vial. The rubber had holes drilled for a reference and auxiliary electrode and a slit cut for a 7 × 70 mm platinum mesh working electrode so that the surface of the mesh faced the working and auxiliary electrodes. Teflon tubing was inserted through a pinhole in the septum for an argon purge and vent. The setup used a CHI112 nonaqueous Ag/AgNO_3_ reference electrode. The auxiliary electrode was formed by removing the silver wire from a CHI112 nonaqueous reference electrode and replacing it with a rolled 7 × 70 mm piece of platinum mesh. The tube was filled with supporting electrolyte. Electrolyses were run on 2 to 3 mL samples, placing the platinum mesh electrode ca. 20 mm into the solution. To avoid the buildup of charge while maximizing current and efficiency, solutions were set to precondition before scanning the potential window with multiple repetitions run. Solutions of **1b** were preconditioned at −2.5 V for 5 seconds and 0 V for 6 seconds and 16–20 repetitions run. Solutions of **3a**,**b** were preconditioned at −2.2 V for 5 seconds and 0 V for 6 seconds with 16 repetitions. The electrolyzed solutions were opened to air or had a chemical oxidant (benzoquinone) added to complete the reduction of the dianion to the LW isomer.

NMR spectroscopy was performed on samples 5 mm NMR tubes (Wilmad) made of clear quartz (photolyzed samples) or amber pyrex (dark samples) on a Varian Mercury or Bruker AvanceIII 400 MHz NMR. ^1^H NMR experiments on **5a** were performed on an argon-purged, ca. 24 mM solution in acetone-*d*_6_. 1D NOE spectra were collected using 400 manually interleaved scans with a 4 s relaxation delay, targeting a single peak per experiment, in a manner similar to that previously reported [21] for photogenerated **4a**,**b**.

### Computational modeling

Predicted ground-state reduction potentials were computed based on the energy difference of the ground-state molecule (S_0_) and its one electron-reduced (D_0_) radical anion and our published correlation of this energy difference with experimental reduction potentials (vs SCE in acetonitrile) [22]. Gas-phase geometries were optimized at the B3LYP/6-31G(d) level of theory. Single point energies were computed at the same level of theory with implicit acetonitrile solvent implemented using the Conductor-like Polarizable Continuum Model with UAKS radii. These computations were performed using the Gaussian 03 software package [25], implemented through the WebMO [26] graphical user interface on the Curie cluster [27] in the Hope College Computational Science & Modeling Laboratory on a single node (a single 2.60 GHz AMD Opteron-252 processor with 8 GB RAM and 250 GB HD).

Calculations of bond length, bond order, and molecular orbitals to rationalize the observed differential photochromic and electrochromic ring-opening of **1** to **2** and **3**, respectively, were performed on the Midwest Undergraduate Computation Chemistry Consortium (MU3C) cluster [28–29]. Computations were performed on a single node (dual Intel X5650 CPU, with 6 cores running at 2.66 GHz) using the Gaussian 09 [30] software package implemented through the WebMO [26] graphical user interface. Restricted open-shell Hartree-Fock (ROHF) theory [31] with the Becke 3, Lee, Yang, and Parr (B3LYP) hybrid functional [32–34] was used for geometry optimizations, molecular orbitals, and bond orders calculations with the 6-31G(d) basis set [35] for open-shell species (T_0_ and D_0_), while conventional B3LYP (with standard Hartree-Fock theory) was used for closed-shell S_0_ calculations. The concerted use of ROHF and B3LYP provides a restricted open-shell B3LYP (DFT) method, which was particularly important to obtaining good bond orders for open-shell (T_0_ and D_0_) species. Bond orders were calculated using the Natural Bond Order (NBO) 3.1 package [36] contained within Gaussian 09. The geometry was first optimized for the S_0_ state in the gas phase. Based on this starting point, geometries for S_0_, T_0_, and the one-electron reduced D_0_ states were then (re)optimized with an implemented acetonitrile conductor-like polarizable continuum model (CPCM) as the self-consistent reaction field (SCRF) [37]. It is from these implicit solvent-optimized geometries that reported bond lengths were derived. Molecular orbital and bond order calculations were subsequently performed on these geometries.

## Supporting Information

File 1 Additional figures and Z-matrices.

File 2 Complete data sets for experimental and computational reduction potentials of all compounds.
