# Supplementary material for: Experimental and computational electrochemistry of quinazolinespirohexadienone molecular switches – differential electrochromic vs photochromic behavior
Source: Beilstein J Org Chem. 2019 Oct 18;15:2473–85. doi: 10.3762/bjoc.15.240 (PMC6808210; doi:10.3762/bjoc.15.240)
Supplement: File 1 — Additional figures and Z-matrices. [file Beilstein_J_Org_Chem-15-2473-s001.pdf]

BEILSTEIN JOURNAL OF ORGANIC CHEMISTRY

Supporting Information

for

Experimental and computational electrochemistry of quinazolinespirohexadienone molecular switches – differential electrochromic vs photochromic behavior

Eric W. Webb, Jonathan P. Moerdyk, Kyndra B. Sluiter, Benjamin J. Pollock, Amy L. Speelman, Eugene J. Lynch, William F. Polik and Jason G. Gillmore

Beilstein J. Org. Chem. **2019,** *15,* 2473–2485. doi:10.3762/bjoc.15.240

Additional figures and Z-matrices

License and Terms: This is a supporting information file under the terms of the Creative Commons Attribution License (http://creativecommons.org/licenses/by/4.0). Please note that the reuse, redistribution and reproduction in particular requires that the authors and source are credited.
The license is subject to the *Beilstein Journal of Organic Chemistry* terms and conditions: (https://www.beilstein-journals.org/bjoc)

Table of contents:

Frontier molecular orbitals for **1b** and **3b**	S1
Geometries for **3a** S_0, T_0, D_0	S2
Z-matrix for **3a** S_0 geometry	S3
Z-matrix for **3a*** T_0 geometry	S8
Z-matrix for **3a**$^{\bullet-}$ D_0 geometry	S13
Excel Spreadsheet with <u>all</u> Experimental and Computational E^{o}_{red} Data	separate file

Figure S1: Frontier molecular orbitals for **1b** and **3b**, cf. Figure 6 (for **1a**). HOMO (MO 97 for **1b**, 101 for **3b**) in red/blue; LUMO (MO 98 for **1b**, 102 for **3b**) in green/yellow.

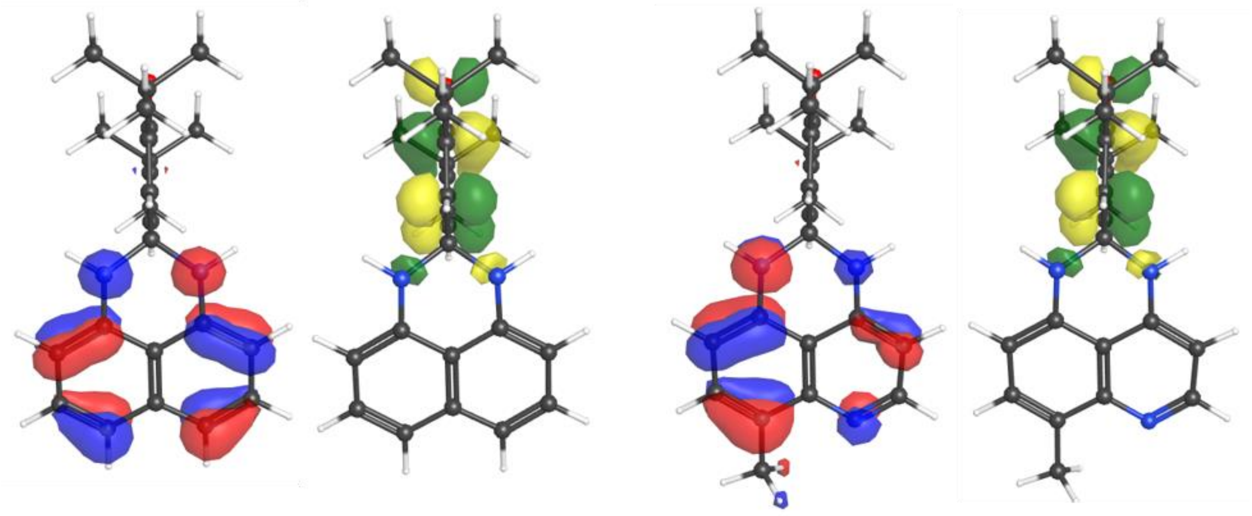

1b MO 97 **1b** MO 98 **3b** MO 101 **3b** MO 102

Figure S2: Geometry visualizations for **3a** S_0, T_0, D_0 (Z-matrices in Figures S3–S5).

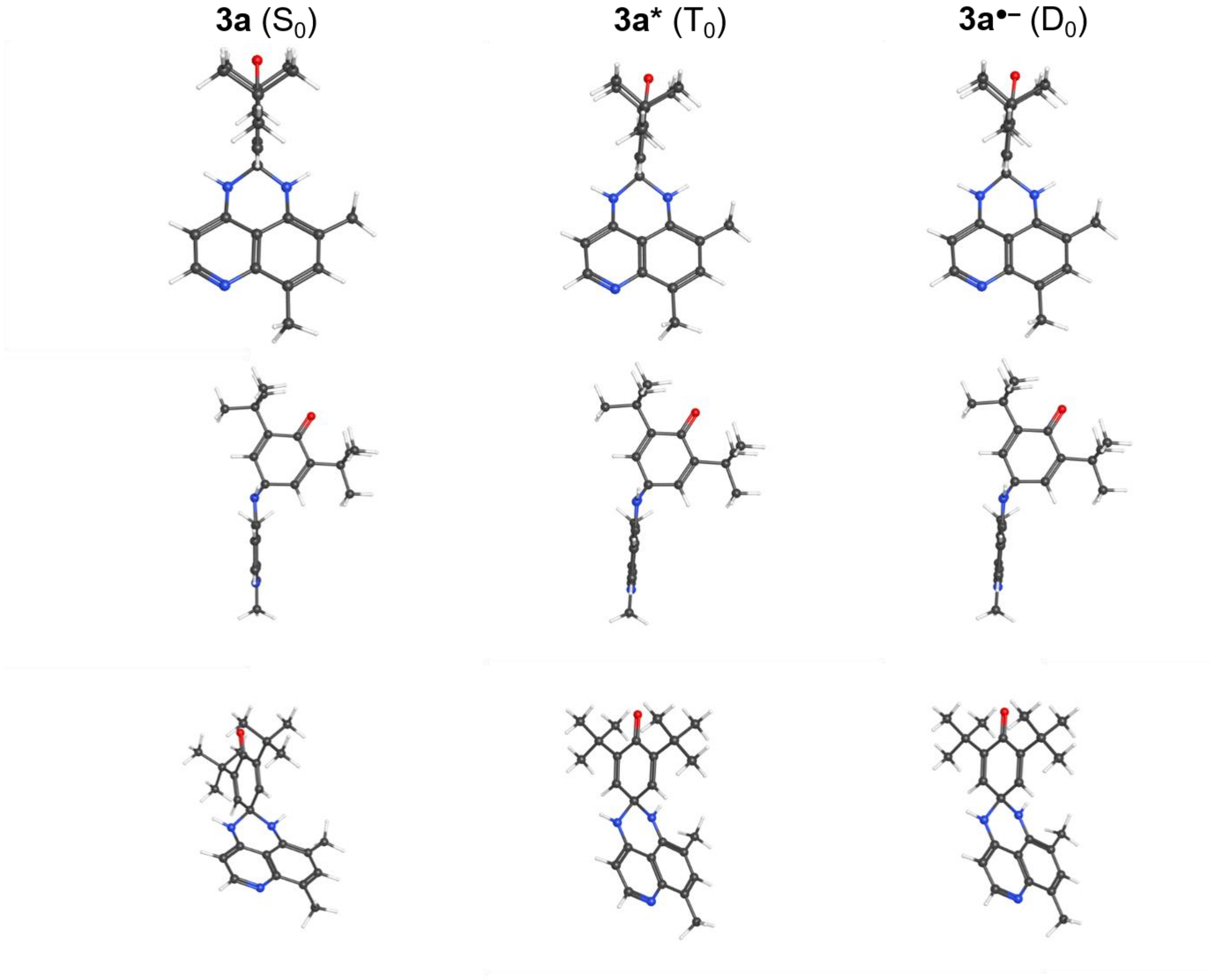

Figure S3: Z-matrix for **3a** S_0 geometry

C						
C	1	B1				
C	2	B2	1	A1		
C	3	B3	2	A2	1	D1
C	4	B4	3	A3	2	D2
C	5	B5	4	A4	3	D3
C	2	B6	3	A5	4	D4
N	7	B7	2	A6	3	D5
C	8	B8	7	A7	2	D6
C	9	B9	8	A8	7	D7
C	6	B10	7	A9	2	D8
N	11	B11	6	A10	7	D9
C	12	B12	11	A11	6	D10
N	5	B13	4	A12	3	D11
H	14	B14	5	A13	4	D12
C	13	B15	12	A14	11	D13
C	16	B16	13	A15	12	D14
C	17	B17	16	A16	13	D15
C	18	B18	17	A17	16	D16
C	19	B19	18	A18	17	D17
H	20	B20	19	A19	18	D18
C	19	B21	18	A20	17	D19
C	22	B22	19	A21	18	D20
H	23	B23	22	A22	19	D21
H	23	B24	22	A23	19	D22
H	23	B25	22	A24	19	D23
C	22	B26	19	A25	18	D24
H	27	B27	22	A26	19	D25
H	27	B28	22	A27	19	D26
H	27	B29	22	A28	19	D27
C	22	B30	19	A29	18	D28
H	31	B31	22	A30	19	D29
H	31	B32	22	A31	19	D30
H	31	B33	22	A32	19	D31
O	18	B34	17	A33	16	D32
C	17	B35	16	A34	13	D33
C	36	B36	17	A35	16	D34
H	37	B37	36	A36	17	D35
H	37	B38	36	A37	17	D36
H	37	B39	36	A38	17	D37
C	36	B40	17	A39	16	D38
H	41	B41	36	A40	17	D39
H	41	B42	36	A41	17	D40
H	41	B43	36	A42	17	D41
C	36	B44	17	A43	16	D42
H	45	B45	36	A44	17	D43
H	45	B46	36	A45	17	D44
H	45	B47	36	A46	17	D45
H	16	B48	13	A47	12	D46
H	12	B49	11	A48	6	D47

H	10	B50	11	A49	6	D48
H	9	B51	10	A50	11	D49
C	4	B52	5	A51	6	D50
H	53	B53	4	A52	5	D51
H	53	B54	4	A53	5	D52
H	53	B55	4	A54	5	D53
H	3	B56	4	A55	5	D54
H	1	B57	2	A56	3	D55
H	1	B58	2	A57	3	D56
H	1	B59	2	A58	3	D57

B1	1.507714864
B2	1.384706967
B3	1.415486447
B4	1.392744841
B5	1.421360869
B6	1.427154868
B7	1.368964415
B8	1.325607513
B9	1.406515462
B10	1.425256515
B11	1.380375493
B12	1.478031950
B13	1.401427077
B14	1.014126111
B15	1.506855615
B16	1.345831092
B17	1.500382686
B18	1.508805062
B19	1.342648379
B20	1.084048132
B21	1.540404757
B22	1.541896378
B23	1.095346550
B24	1.096030777
B25	1.096012336
B26	1.549126791
B27	1.096605485
B28	1.090852705
B29	1.096705055
B30	1.549075264
B31	1.096628923
B32	1.096692426
B33	1.090816313
B34	1.232025701
B35	1.541259530
B36	1.548931562
B37	1.096715718
B38	1.096750514
B39	1.090960800
B40	1.541734658
B41	1.096142838

B42 1.096144838
B43 1.095398093
B44 1.548946452
B45 1.091009410
B46 1.096718298
B47 1.096736414
B48 1.084958401
B49 1.013857131
B50 1.085795090
B51 1.090327199
B52 1.509665060
B53 1.093592858
B54 1.098471473
B55 1.099455765
B56 1.088565215
B57 1.094616254
B58 1.096618657
B59 1.096607790
A1 121.5815408
A2 124.3078648
A3 117.9077027
A4 119.7931322
A5 118.0033891
A6 119.3499220
A7 117.1484875
A8 125.5661628
A9 118.8665484
A10 118.4101388
A11 119.6651252
A12 122.3420238
A13 114.4535637
A14 110.4954287
A15 125.2462631
A16 118.3352000
A17 119.4413496
A18 118.6630811
A19 121.9128556
A20 119.1245483
A21 111.5152804
A22 109.0498628
A23 112.0941300
A24 112.1012467
A25 110.0331839
A26 109.5508977
A27 111.6567471
A28 110.7291785
A29 110.0214956
A30 109.5613181
A31 110.7207993
A32 111.6360485
A33 120.5499632
A34 122.5059470

A35 110.1256249
A36 110.7242654
A37 109.6395953
A38 111.6010940
A39 111.4733841
A40 112.1027180
A41 112.1008298
A42 109.0951206
A43 110.0439993
A44 111.6150350
A45 109.6528093
A46 110.7271529
A47 113.8666742
A48 115.7393978
A49 121.2516884
A50 118.3791219
A51 121.1737880
A52 110.8548870
A53 111.9249185
A54 112.0605803
A55 117.2974209
A56 110.7468408
A57 111.5819223
A58 111.5856111
D1 179.8688604
D2 0.073717124
D3 0.059429412
D4 0.280819647
D5 178.8374014
D6 -178.8761467
D7 -0.550899975
D8 179.1666850
D9 -177.6038485
D10 -25.17718236
D11 -176.1437297
D12 -20.24565334
D13 -74.03062351
D14 -121.1533832
D15 0.485576636
D16 -0.727770392
D17 0.565428406
D18 -179.9499012
D19 -179.4192028
D20 179.7266253
D21 -179.9457091
D22 -60.99582158
D23 61.11635327
D24 60.45274435
D25 178.5164866
D26 -61.32780655
D27 59.78896897
D28 -60.98965103

D29	-178.6798208
D30	-59.94292155
D31	61.15640390
D32	179.3455007
D33	-179.4797636
D34	-119.1599387
D35	59.85045422
D36	178.6411535
D37	-61.20375290
D38	0.163112706
D39	-61.13398226
D40	60.96400255
D41	179.9179895
D42	119.4239167
D43	61.07893460
D44	-178.7690157
D45	-59.96716034
D46	59.20094849
D47	-166.6103324
D48	179.9283389
D49	179.6445418
D50	179.4746696
D51	179.2138335
D52	-60.71766739
D53	59.40065809
D54	-179.8142697
D55	0.421590815
D56	121.0026485
D57	-120.1664666

Figure S4: Z-matrix for **3a*** T_0 geometry

```
C
C     1    B1
C     2    B2   1    A1
C     3    B3   2    A2   1    D1
C     4    B4   3    A3   2    D2
C     5    B5   4    A4   3    D3
C     2    B6   3    A5   4    D4
N     7    B7   2    A6   3    D5
C     8    B8   7    A7   2    D6
C     9    B9   8    A8   7    D7
C     6   B10   7    A9   2    D8
N    11   B11   6   A10   7    D9
C    12   B12  11   A11   6   D10
N     5   B13   4   A12   3   D11
H    14   B14   5   A13   4   D12
C    13   B15  12   A14  11   D13
C    16   B16  13   A15  12   D14
C    17   B17  16   A16  13   D15
C    18   B18  17   A17  16   D16
C    19   B19  18   A18  17   D17
H    20   B20  19   A19  18   D18
C    19   B21  18   A20  17   D19
C    22   B22  19   A21  18   D20
H    23   B23  22   A22  19   D21
H    23   B24  22   A23  19   D22
H    23   B25  22   A24  19   D23
C    22   B26  19   A25  18   D24
H    27   B27  22   A26  19   D25
H    27   B28  22   A27  19   D26
H    27   B29  22   A28  19   D27
C    22   B30  19   A29  18   D28
H    31   B31  22   A30  19   D29
H    31   B32  22   A31  19   D30
H    31   B33  22   A32  19   D31
O    18   B34  17   A33  16   D32
C    17   B35  16   A34  13   D33
C    36   B36  17   A35  16   D34
H    37   B37  36   A36  17   D35
H    37   B38  36   A37  17   D36
H    37   B39  36   A38  17   D37
C    36   B40  17   A39  16   D38
H    41   B41  36   A40  17   D39
H    41   B42  36   A41  17   D40
H    41   B43  36   A42  17   D41
C    36   B44  17   A43  16   D42
H    45   B45  36   A44  17   D43
H    45   B46  36   A45  17   D44
H    45   B47  36   A46  17   D45
H    16   B48  13   A47  12   D46
H    12   B49  11   A48   6   D47
```

H	10	B50	11	A49	6	D48
H	9	B51	10	A50	11	D49
C	4	B52	5	A51	6	D50
H	53	B53	4	A52	5	D51
H	53	B54	4	A53	5	D52
H	53	B55	4	A54	5	D53
H	3	B56	4	A55	5	D54
H	1	B57	2	A56	3	D55
H	1	B58	2	A57	3	D56
H	1	B59	2	A58	3	D57

B1	1.497699881
B2	1.400954065
B3	1.392470012
B4	1.438856168
B5	1.427144140
B6	1.443339376
B7	1.351240087
B8	1.342532677
B9	1.390708208
B10	1.419887507
B11	1.356818656
B12	1.503044561
B13	1.340057444
B14	1.014563546
B15	1.474937334
B16	1.377551016
B17	1.465413712
B18	1.473730811
B19	1.368199968
B20	1.084022277
B21	1.543710020
B22	1.541954025
B23	1.096285670
B24	1.096203708
B25	1.096220443
B26	1.548536243
B27	1.097893858
B28	1.090633540
B29	1.097497587
B30	1.548494331
B31	1.097782799
B32	1.097435803
B33	1.090721706
B34	1.279192665
B35	1.543165056
B36	1.548629436
B37	1.097469029
B38	1.097854815
B39	1.090767113
B40	1.542141856
B41	1.096320299

B42 1.096286606
B43 1.096348375
B44 1.548910989
B45 1.090805061
B46 1.097881951
B47 1.097442778
B48 1.084579436
B49 1.013883428
B50 1.085302755
B51 1.088452065
B52 1.501756257
B53 1.092186828
B54 1.098052299
B55 1.098154488
B56 1.086728179
B57 1.092789226
B58 1.096528013
B59 1.096388387
A1 121.1548277
A2 123.9275371
A3 117.6842136
A4 119.8271312
A5 118.8274997
A6 118.7470780
A7 116.7503921
A8 125.3714516
A9 119.5677231
A10 119.2181369
A11 123.2846523
A12 122.3060480
A13 118.9824231
A14 112.9397980
A15 124.1947018
A16 120.4297356
A17 117.1948005
A18 120.6108975
A19 121.5336349
A20 119.2132965
A21 112.7684274
A22 109.1867658
A23 112.1346488
A24 112.1463128
A25 109.6626565
A26 110.0861550
A27 110.5597141
A28 110.6408447
A29 109.7043151
A30 110.1084313
A31 110.6176106
A32 110.6265352
A33 121.5358168
A34 120.3023730

A35 109.8047738
A36 110.6177527
A37 110.1121727
A38 110.5420490
A39 112.8454051
A40 112.1401156
A41 112.2275409
A42 109.1537368
A43 109.5152372
A44 110.5077959
A45 110.0908598
A46 110.6555997
A47 114.9039576
A48 117.1409690
A49 120.7122143
A50 118.9193010
A51 120.6307256
A52 110.7268849
A53 111.5691498
A54 111.5469220
A55 117.9282410
A56 111.2081594
A57 110.8206718
A58 110.8438868
D1 179.8607031
D2 0.603998590
D3 -0.996760789
D4 0.525620716
D5 177.9244137
D6 -178.2290537
D7 -0.034774835
D8 177.8949492
D9 -177.2629242
D10 -25.67004328
D11 178.9329076
D12 -2.040850255
D13 -76.87672102
D14 -130.6714857
D15 1.150512501
D16 2.755064955
D17 -2.550166239
D18 179.4719161
D19 176.8223958
D20 -179.1289578
D21 179.8605046
D22 -61.11494832
D23 60.81884539
D24 61.15129961
D25 -179.0820462
D26 -58.52031422
D27 61.97645096
D28 -59.41377415

```
D29  178.8006567
D30 -62.23414336
D31  58.28219182
D32 -178.2723737
D33 -179.3719086
D34 -120.7409455
D35  62.37521503
D36 -178.6736445
D37 -58.13963088
D38 -0.953068752
D39 -60.55622385
D40  61.44197712
D41 -179.5609160
D42  118.7663335
D43  58.48133577
D44  179.0305199
D45 -61.97709265
D46  50.53367599
D47 -174.3370818
D48  179.9687080
D49  179.5408735
D50  179.5650747
D51 -179.5733987
D52 -59.48237068
D53  60.43781312
D54 -179.4915275
D55  0.349819572
D56  121.5218253
D57 -120.9121702
```

Figure S5: Z-matrix for **3a•⁻** D_0 geometry

```
C
C     1    B1
C     2    B2   1    A1
C     3    B3   2    A2   1    D1
C     4    B4   3    A3   2    D2
C     5    B5   4    A4   3    D3
C     2    B6   3    A5   4    D4
N     7    B7   2    A6   3    D5
C     8    B8   7    A7   2    D6
C     9    B9   8    A8   7    D7
C     6   B10   7    A9   2    D8
N    11   B11   6   A10   7    D9
C    12   B12  11   A11   6   D10
N     5   B13   4   A12   3   D11
H    14   B14   5   A13   4   D12
C    13   B15  12   A14  11   D13
C    16   B16  13   A15  12   D14
C    17   B17  16   A16  13   D15
C    18   B18  17   A17  16   D16
C    19   B19  18   A18  17   D17
H    20   B20  19   A19  18   D18
C    19   B21  18   A20  17   D19
C    22   B22  19   A21  18   D20
H    23   B23  22   A22  19   D21
H    23   B24  22   A23  19   D22
H    23   B25  22   A24  19   D23
C    22   B26  19   A25  18   D24
H    27   B27  22   A26  19   D25
H    27   B28  22   A27  19   D26
H    27   B29  22   A28  19   D27
C    22   B30  19   A29  18   D28
H    31   B31  22   A30  19   D29
H    31   B32  22   A31  19   D30
H    31   B33  22   A32  19   D31
O    18   B34  17   A33  16   D32
C    17   B35  16   A34  13   D33
C    36   B36  17   A35  16   D34
H    37   B37  36   A36  17   D35
H    37   B38  36   A37  17   D36
H    37   B39  36   A38  17   D37
C    36   B40  17   A39  16   D38
H    41   B41  36   A40  17   D39
H    41   B42  36   A41  17   D40
H    41   B43  36   A42  17   D41
C    36   B44  17   A43  16   D42
H    45   B45  36   A44  17   D43
H    45   B46  36   A45  17   D44
H    45   B47  36   A46  17   D45
H    16   B48  13   A47  12   D46
H    12   B49  11   A48   6   D47
```

H	10	B50	11	A49	6	D48
H	9	B51	10	A50	11	D49
C	4	B52	5	A51	6	D50
H	53	B53	4	A52	5	D51
H	53	B54	4	A53	5	D52
H	53	B55	4	A54	5	D53
H	3	B56	4	A55	5	D54
H	1	B57	2	A56	3	D55
H	1	B58	2	A57	3	D56
H	1	B59	2	A58	3	D57

B1	1.508208404
B2	1.386876514
B3	1.412426751
B4	1.398918728
B5	1.424123508
B6	1.426596979
B7	1.371280754
B8	1.328700515
B9	1.402458824
B10	1.428248178
B11	1.370148349
B12	1.512831509
B13	1.392019002
B14	1.014220050
B15	1.491043370
B16	1.371112953
B17	1.467640586
B18	1.468809880
B19	1.370723310
B20	1.084601830
B21	1.546057105
B22	1.542184095
B23	1.096964011
B24	1.096311566
B25	1.096342082
B26	1.548049312
B27	1.098612737
B28	1.090909104
B29	1.097877088
B30	1.547986234
B31	1.098624084
B32	1.097834195
B33	1.090830769
B34	1.288767918
B35	1.546329998
B36	1.547739703
B37	1.097871589
B38	1.098617761
B39	1.090863769
B40	1.542156543
B41	1.096405172

B42 1.096413979
B43 1.097048899
B44 1.547993031
B45 1.090930470
B46 1.098592203
B47 1.097836063
B48 1.085349220
B49 1.014444936
B50 1.086343904
B51 1.091331382
B52 1.509736254
B53 1.094177990
B54 1.099178922
B55 1.099849789
B56 1.089383207
B57 1.095228646
B58 1.097218708
B59 1.097191720
A1 121.5448439
A2 124.3627355
A3 118.1232414
A4 119.3081455
A5 117.9895739
A6 119.2439775
A7 116.8461265
A8 125.8098114
A9 119.1161835
A10 118.4866209
A11 120.8107184
A12 122.7645666
A13 114.1118681
A14 111.5675833
A15 124.9074800
A16 120.6651895
A17 116.9637451
A18 120.8845346
A19 121.3404955
A20 119.0056539
A21 113.0191175
A22 109.2787562
A23 112.1243106
A24 112.1519137
A25 109.7026045
A26 110.4609217
A27 110.2131970
A28 110.5508576
A29 109.7024071
A30 110.4614825
A31 110.5525937
A32 110.2360008
A33 121.5885705
A34 120.3570942

```
A35  109.6732485
A36  110.5959816
A37  110.4930133
A38  110.0473656
A39  112.9810771
A40  112.1247951
A41  112.1619868
A42  109.3231531
A43  109.7683804
A44  110.1915886
A45  110.4406261
A46  110.5721859
A47  114.5248367
A48  115.4240336
A49  120.9767381
A50  118.3511842
A51  120.8904554
A52  110.9241274
A53  112.0032142
A54  112.1476221
A55  117.3549498
A56  110.7613764
A57  111.7776607
A58  111.7829136
D1  179.9962626
D2 -0.213208317
D3  0.479557072
D4  0.201011111
D5  179.3727990
D6 -179.4507170
D7 -0.388396700
D8  179.5061322
D9 -177.9334501
D10 -27.12738495
D11 -176.3682020
D12 -19.58850194
D13 -72.66325040
D14 -121.9105413
D15 -0.047561689
D16 -0.497821226
D17  0.542217647
D18 -179.8021946
D19 -179.4294257
D20  179.6364383
D21 -179.7287191
D22 -60.60320952
D23  61.13409520
D24  59.87419119
D25 -178.3891986
D26 -57.69515867
D27  62.55232243
D28 -60.59566251
```

D29	178.6325769
D30	-62.28935006
D31	57.93109778
D32	179.5936975
D33	179.9709640
D34	-119.7912988
D35	62.54872175
D36	-178.2830654
D37	-57.61930946
D38	-0.018369053
D39	-60.87891356
D40	60.88006076
D41	-179.9884103
D42	119.8080104
D43	57.68872575
D44	178.3200041
D45	-62.57702881
D46	58.06945909
D47	-166.6027576
D48	179.7268996
D49	179.9647304
D50	179.7300178
D51	-179.8223282
D52	-59.63627907
D53	60.24522696
D54	179.8673068
D55	0.097316044
D56	120.6133134
D57	-120.4797099
